# Preliminary study of the effect of gut microbiota on the development of prostatitis

**DOI:** 10.1186/s12920-024-01812-y

**Published:** 2024-01-25

**Authors:** Cheng Shen, Zhan Chen, Wei Zhang, Xinfeng Chen, Bing Zheng, Chunmei Shi

**Affiliations:** 1 Department of Urology, Affiliated Hospital 2 of Nantong University, Nantong, 226001 China; 2 Medical Research Center, Affiliated Hospital 2 of Nantong University, Nantong, China

**Keywords:** Prostatitis, Gut microbiota, Mendelian randomization, Causal effects, Risk factors

## Abstract

**Background:**

Dysbacteriosis of intestinal tract may cause systemic inflammation, making distant anatomical locations more susceptible to illness. Recent research has demonstrated that the microbiome can affect both prostatitis and the inflammation of the prostate that is linked to prostate cancer. It is still unclear, though, whether this relationship indicates causation. We conducted a Mendelian randomization investigation on two samples to fully uncover gut microbiota’s potential genetic causal role in prostatitis.

**Method:**

Prostatitis (1859 prostatitis cases and 72,799 controls) was utilized as the outcome, while SNPs highly linked with 196 microbial taxa (18 340 people) were chosen as instrumental factors. Random effects, inverse variance weighting, weighted medians, and MR-Egger were used to analyze causal effects. The Cochran’s Q test, funnel plot, leave-one-out analysis, and MR-Egger intercept test were all used in the sensitivity analysis.

**Results:**

A causal effect in lowering the incidence of prostatitis is anticipated for five gut microorganisms (*Methanobacteria*, *Methanobacteriaceae*, *Erysipelatoclostridium*, *Parasutterella*, and *Slackia*; *P* < 0.05). Four gut bacteria, including *Faecalibacterium*, *LachnospiraceaeUCG004*, *Sutterella*, and *Gastranaerophilales*, are predicted to play a causal role in increasing the risk of prostatitis (*P* < 0.05). There were no discernible estimates of pleiotropy or heterogeneity.

**Conclusion:**

Our investigation established the genetic links between nine gut microorganisms and prostatitis, which may offer fresh perspectives and a theoretical framework for the future prevention and management of prostatitis.

**Supplementary Information:**

The online version contains supplementary material available at 10.1186/s12920-024-01812-y.

## Introduction

Prostatitis, a condition that affects men, is mainly characterized by inflammation or swelling of the prostate gland. The National Institutes of Health (NIH) consensus classification for prostatitis syndrome includes four categories, and Table 1 shows the presence of an inflammatory response and symptoms in each category [1]. Up to half of all men are thought to have experienced prostatitis symptoms at some point in their lives [2]. Prostatitis accounted for 2 million office visits annually in the USA in the early 1990s [3], almost matching the number of appointments for benign prostatic hypertrophy (BPH). It ranks as the third most prevalent urological diagnostic for males over 50 and the most common urological diagnosis for men under 50 [3]. Chronic prostatitis has been shown to have an effect on a patient’s quality of life that is comparable to that of angina, myocardial infarction, or Crohn’s disease [4]. Just with BPH and cancer, the other two central prostatic disorders, prostatitis is a significant health concern [5].

**Table 1:** Consensus classification of prostatitis syndrome

Name	Classification	Presence of inflammatory reaction	symptoms
Acute bacterial prostatitis	Type I	Yes	Yes
Chronic bacterial prostatitis	Type II	Yes	Yes
Chronic pelvic pain syndrome (CPPS)	Type IIIA	Yes	Yes
	Type IIIB	No	Yes
Asymptomatic in¯ammatory prostatitis	Type IV	Yes	No

In clinical practice, antibiotics, alpha-blockers, phytotherapy, and hormone therapy are frequently employed [6]. Effective chronic nonbacterial prostatitis (CNP) treatment, however, remains a significant issue for medical professionals because of the etiology’s lingering controversy. Alpha-blockers, antibiotics, and their combination did not succeed in lowering the chronic prostatitis symptom score, according to a meta-analysis [7]. According to a recent study, Poria cocos polysaccharides (PPs) can alter the gut microbiota, which enhances CNP [8]. Previous research has shown that CNP animals and patients experience intestinal dysbacteriosis [9, 10], but no studies have looked into the causal relationship between intestinal microbiota and prostatitis or focused on the exact role of specific intestinal microbiota taxa on prostatitis. We hypothesized that changes in the gut microbiota composition cause prostatitis in light of the results mentioned earlier. Studies on the role of particular intestinal microbiota against prostatitis are currently scarce since intestinal microbiota is a complex microbial community encompassing numerous groups. Overall, it is crucial for clinical practice in treating prostatitis to confirm the causal link of this interaction and which microbial taxa are most important.

Mendelian randomization (MR) is another approach to account for observed bias, using genetic variants, typically single nucleotide polymorphisms (SNPs), as instrumental variables (IVs) to detect causal relationships between exposure and disease outcomes [11]. The IVs associated with the exposure will have a proportionate impact on the outcome if there is a causal relationship between the direction and the outcome [12]. The probability of each allele being inherited at random by a person is the same, which makes MR research comparable to randomized controlled trials (RCTs) [13]. MR is more effective than traditional observational research in avoiding reverse causal relationships and confounding factors because genetic variants are typically unrelated [14]. Recent genetic studies have shown host genetic variants influence gut microbiota composition [15]. Thus, these findings allow us to employ MR methods to infer the causal relationship between gut microbiota and prostatitis.

To determine the potential causative role of 196 microbial taxa species on prostatitis, we conducted a two-sample MR analysis based on widely accessible large-scale GWAS data on the gut microbiome and the condition. Finally, even though most of these studies have not yet involved the urological sector, we validate the function of particular gut bacteria in raising or lowering the risk of prostatitis. Therefore, our findings not only broaden the taxonomy of gut microorganisms linked to prostatitis but also highlight their unique causal relationship with the condition, offering fresh approaches for its therapeutic management.

## Materials and methods

### Study procedures

**Fig. 1:**
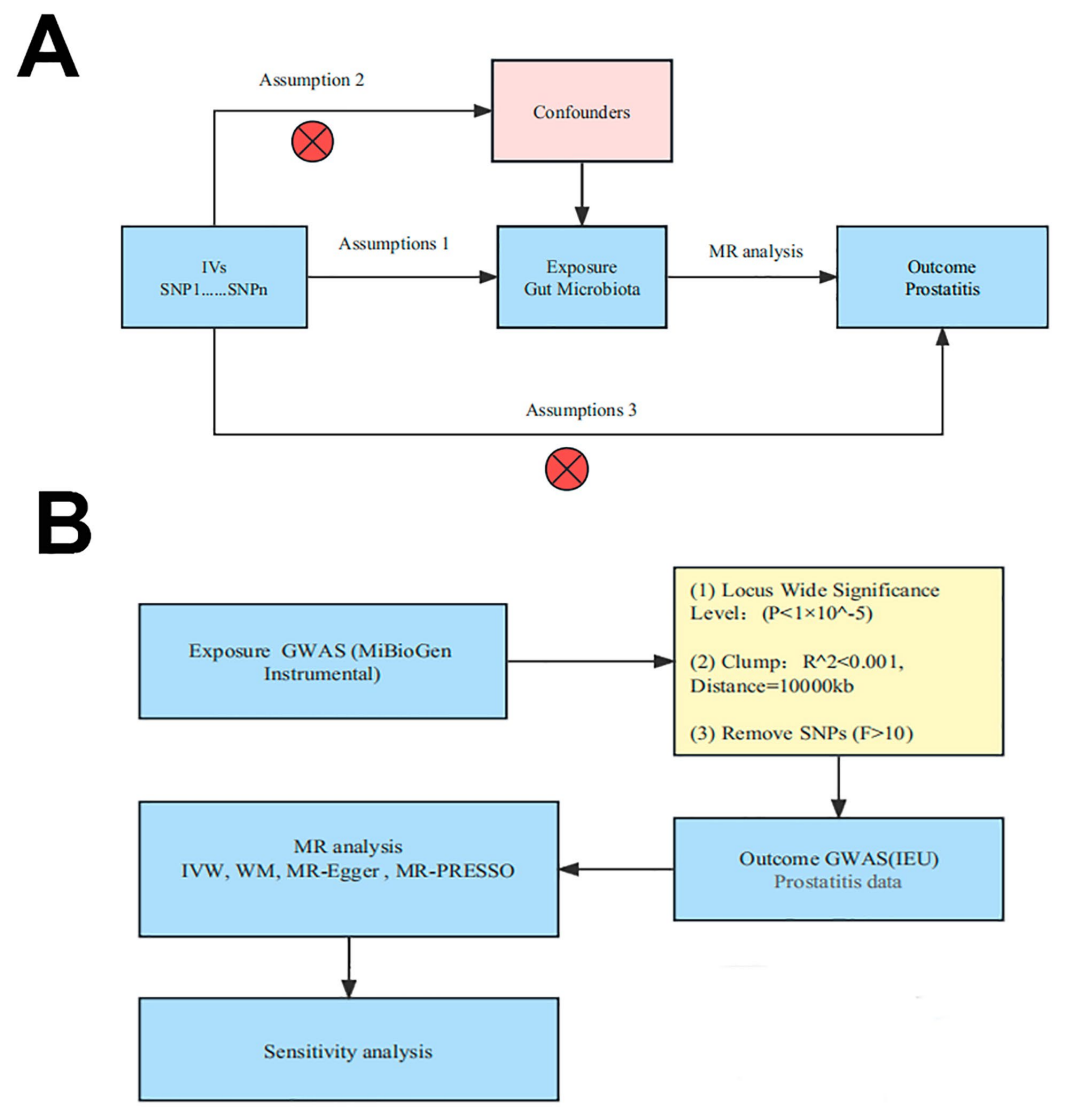
Schematic representation of causal relationship between gut microbiota and prostatitis by MR analysis **(A)** Mendelian randomization is based on three assumptions. **(B)** Flowchart of this Mendelian randomization study. GWAS, Genome Wide Association Studies; IV, Instrumental variable; SNP, single nucleotide polymorphism; MR, Mendelian randomization; IVW, Inverse-variance weighted; WM, Weighted median

In this work, we conducted a thorough MR analysis to identify the causal link between 196 intestinal microbial taxa and prostatitis. Figure 1A shows the structure of our study design. 196 microbial taxa taxonomic exposure that led to prostatitis was the subject of investigation. It was possible to get and reconcile the data from the findings with the exposed instrument variables. Then, sensitivity analysis and MR analysis (using three different methodologies) were carried out. Three hypotheses should guide carefully constructed MRs: (1) the genetics variant (human genetics) was strongly correlated with the exposure of interest (the gut microbiota); (2) genetic variation is not correlated with a potential risk factor for the outcome (prostatitis) (*P* < 1 × 10^− 5^); and (3) genetic variation only influences the outcome through exposure [13]. All of these presumptions were adequately addressed in our analysis. To determine the strength of the first hypothesis, we isolated vital tool variables and computed their F statistics. The study design satisfies the second hypothesis since SNPs on each chromosome were randomly assigned during meiosis by Mendelian’s second law [16]. Finally, we used MR-Egger intercept analysis to test for pleiotropy in the third hypothesis.

### Acquisition of data sources

Exposure data (gut microbiota) was obtained from MiBioGen [17] (data link: https://mibiogen.gcc.rug.nl), which included 16 S ribosomal RNA gene sequencing profiles and genotyping data from 18,340 individuals, 211 taxons, and 122,110 variant loci [18], all of which were standardized for age, sex(excluding female), study-specific covariates, and the top genetic principal components of population stratification [15]. Excluding 15 unknown groupings left 196 microbial taxa, including 9 phyla, 16 classes, 20 orders, 32 families, and 119 genera (Supplemental Table 1).

First, to acquire more association results, we used exposure data with *P* < 10^− 5^ rather than data with *P* < 10^− 8^ because few of the gut microbiota gene loci revealed by GWAS achieved genome-wide significance levels (*P* < 10^− 8^) [19]. Second, we picked a cluster distance of 10,000 kb and a r^2^ < 0.001 to rule out linkage disequilibrium between the genetic tools. For each SNP, we computed the F and R^2^ values using the formula below to examine the impact on gut microbiota: F = [R^2^ × (N-2)]/(1-R^2^), R^2^ = [2 × β^2^ × EAF × (1-EAF)]/[2 × β^2^ × EAF × (1-EAF) + 2 × SE^2^ × N × EAF × (1-EAF)] [19–21]. Here, N stands for sample size, EAF for effector allele frequency, and SE for estimated effect size on the gut microbiota of SNPs [19–21]. Due to their insufficient validity [22], we eliminated SNPs having F values of less than 10 (Fig. 1B).

Data collection for the result (prostatitis): We downloaded the IEU Open GWAS project (data link: https://gwas.mrcieu.ac.uk/), GWAS data for prostatitis (id: finn-b-N14PROSTATITIS), and generated pooled level data for all GWAS related to prostatitis. After obtaining the SNP information for exposure and outcome, we harmonized the data for further analysis.

The GWAS summary statistics used in this MR investigation were carried out with ethical permission for each GWAS. Published studies and online summary statistics that are accessible to the general public were used. These de-identified summary data are all freely downloadable and permissible for unlimited usage.

### Mendelian randomization analysis and sensitivity analysis

Methods for sensitivity and MR analyses were consistent with those employed in earlier studies [12]. Since random effects inverse variance weighting (IVW) is the most reliable analysis and may offer conservative estimates even in the face of heterogeneity, it was chosen as the primary analysis. In addition, we conducted tests for Mendelian random pleiotropic residuals and outliers (MR-PRESSO), weighted median (WM), MR-Egger regression, and robustness validation. Tests for unbalanced pleiotropy and significant heterogeneity are available with MR-Egger regression. When pleiotropy is present, estimates based on the MR-Egger method are more convincing than those based on the IVW method [23]. When the horizontal pleiotropy-provided weighted variance is at least 50% genuine, WM estimation can produce reliable estimates of impact [24]. In a nutshell, IVW offered substantial estimates in the same general direction as those offered by WM and MR-Egger and were therefore regarded as essential estimates.

### Statistics

All statistical testing was conducted in R (version 4.2.1) using the “TwoSampleMR” package (version 0.5.6). The significance level was established at a two-sided *P* < 0.05. All estimates were shown as odds ratios (ORs) for the relevant exposure’s additional standard deviation (SD).

## Results

### Overview of instrumental variables (IVs) in taxa

Genome-wide significance threshold (*P* < 10^− 5^) screening, Linkage disequilibrium testing (LD testing), coordination, MR-PRESSO testing, and F-statistic validation were used to identify several SNPs ranging from 6 to 15 as proxies for each of the 196 microbial taxa. All SNPs identified by MR-PRESSO as outliers (global test: *P* > 0.05) were eliminated. F values above 10 were present for all preserved SNPs, showing an adequate association between IVs and associated microbial taxa. Table 2 displays the final retained SNP list and related data. In addition, the findings of the Mendelian randomization analysis of all 196 microbial taxa with prostatitis are shown in Supplemental Tables 2, and details of all instrumental factors are presented in Supplemental Table 3, respectively.

**Table 2:** MR estimates for the relationship between genetically instrumented gut microbiota and Prostatitis

Classification		nSNP	SE	*P*-value	OR (95% CI)		Pleiotropy			Heterogeneity		MR-PRESSO
							Egger intercept	SE	*P*-value	Q	*P*-value	
Category												
class	*Methanobacteria.*id.119	9	0.108	0.001	0.692(0.560–0.855)		-0.038	0.068	0.586	4.709	0.788	0.586
family	*Methanobacteriaceae.*id.121	9	0,108	0.001	0.692(0.560–0.855)		-0.039	0.067	0.586	4.709	0.788	0.586
Genus	*Erysipelatoclostridium*.id.11,381	15	0.132	0.011	0.714(0.551–0.925)		-0.027	0.042	0.536	14.871	0.387	0.536
	*Faecalibacterium.*id.2057	10	0.196	0.018	1.591 (1.082–2.340)		0.026	0.055	0.641	11.099	0.435	0.15
·												
	*LachnospiraceaeUCG004*.id.11,324	12	0.183	0.007	1.639 (1.147–2.343)		0.039	0.048	0.440	7.332	0.711	0.440
	*Parasutterella.*id.2892	14	0.132	0.023	0.740 (0.571–0.959)		0.003	0.029	0.925	7.144	0.894	0.915
	*Slackia*.id.825	6	0.170	0.030	0.690 (0.494–0.964)		0.151	0.109	0.239	3.443	0.631	0.239
	*Sutterella.*id.2896	12	0.168	0.007	1.578 (1.135–2.194)		-0.058	0.048	0.257	7.601	0.748	0.257
Order	*Gastranaerophilales.*id.1591	9	0.147	0.008	1.476 (1.104–1.972)		-0.020	0.053	0.71	10.164	0.253	0.711

MR-PRESSO, Mendelian randomization pleiotropy residual sum and outlier; SNPs, single nucleotide polymorphisms; CI, confidence interval; OR, odds ratio; Q, heterogeneity statistic Q; SE, Standard Error;

### Association of intestinal microbial taxa with prostatitis

We found a positive association between prostatitis risk and four gut microorganisms: *Faecalibacterium* (OR = 1.591, 95% CI: 1.082–2.340, *p* = 0.018), *LachnospiraceaeUCG004* (OR = 1.639, 95% CI: 1.147–2.343, *p* = 0.007), *Sutterella* (OR = 1.578, 95% CI: 1.135–2.194, *p* = 0.007), and *Gastranaerophilales* (OR = 1.476, 95% CI: (1.104–1.972), *p* = 0.008), this implies that these bacteria may raise the risk of prostatitis. Sensitivity analysis failed to find any proof of pleiotropy at any level. Four groups of the gut microbiota underwent weighted median analysis, and the directionality of these results agreed with IVW (Table 3). We further classified the staining properties of these four microbial taxa (Table 4).

**Table 3:** The correlations between the risks of prostatitis in the IEU database and nine gut microbial genera that are genetically defined

Outcome	Expoure	Method	nSNP	OR	or_lci95	or_uci95	pval
Prostatitis	class.*Methanobacteria.*id.119	Weighted median	9	0.731	0.547	0.977	0.034
		Inverse variance weighted	9	0.692	0.560	0.855	0.001
	family.*Methanobacteriaceae.*id.121	Weighted median	9	0.731	0.548	0.974	0.032
		Inverse variance weighted	9	0.691	0.560	0.855	0.001
	genus.*Erysipelatoclostridium*.id.11,381	Weighted median	15	0.754	0.525	1.083	0.127
		Inverse variance weighted	15	0.714	0.551	0.925	0.011
	genus.*Faecalibacterium*.id.2057	Weighted median	10	1.544	0.944	2.523	0.083
		Inverse variance weighted	10	1.591	1.082	2.340	0.018
	genus.*LachnospiraceaeUCG004*.id.11,324	Weighted median	12	1.893	1.179	3.039	0.008
		Inverse variance weighted	12	1.639	1.147	2.343	0.007
	genus.*Parasutterella*.id.2892	Weighted median	14	0.754	0.526	1.081	0.124
		Inverse variance weighted	14	0.740	0.571	0.959	0.023
	genus.*Slackia*.id.825	Weighted median	6	0.699	0.456	1.072	0.101
		Inverse variance weighted	6	0.690	0.494	0.964	0.030
	genus.*Sutterella*.id.2896	Weighted median	12	1.626	1.037	2.549	0.034
		Inverse variance weighted	12	1.578	1.135	2.194	0.007
	order.Gastranaerophilales.id.1591	Weighted median	9	1.534	1.082	2.177	0.016
		Inverse variance weighted	9	1.476	1.104	1.972	0.008

**Table 4:** Categorization of staining properties for high-risk taxa

Categorization	Gram-positive bacteria	Gram-negative Bacteria	Aerobic Bacteria	Anaerobic Bacteria	Common human microorganism	Common microorganism
*Faecalibacterium*	Positive		Positive		Positive	
*LachnospiraceaeUCG004*	Positive			Positive	Positive	
*Sutterella*		Positive		Positive	Positive	
*Gastranaerophilales*	Positive			Positive	Positive	Positive

On the other hand, we found that five gut microorganisms were associated with a decreased risk of prostatitis: *Faecalibacterium* (OR = 0.692, 95% CI: 0.560–0.855, *p* = 0.001), *Methanobacteriaceae* (OR = 0.692, 95% CI: 0.560–0.855, *Erysipelatoclostridium* (OR = 0.714, 95% CI: (0.551–0.925), *p* = 0.036), *Parasutterella* (OR = 0.740, 95% CI: (0.571–0.959), *p* = 0.023). *Slackia* (OR = 0.690, 95% CI: (0.494–0.964), *p* = 0.030) indicates that these bacteria might have a preventative impact against prostatitis. Sensitivity analysis failed to find proof of pleiotropy, no matter what degree. A weighted median examination of five groups of gut microorganisms produced directionality in forest plots consistent with IVW (Table 2). The leave-one-out assay revealed no aberrant SNPs. In Supplementary Figure, scatter plots and the results of the leave-one-out test are displayed. The findings above show a consistent genetically based causal link between the gut microbiota and prostatitis.

## Discussion

Currently, antimicrobial agents are effective in improving CNP symptoms in patients. For example, nearly half of CNP patients can be enhanced by fluoroquinolone therapy [25]. Roxithromycin and ciprofloxacin have also been demonstrated to improve CNP symptoms significantly [26]. Alpha-blockers, such as terazosin, alfuzosin, and tamsulosin, are also widely used to treat CNP [6], and the combination of alpha-blockers with antibiotics seems more effective [27]. However, the effectiveness of alpha-blockers is influenced by patient age [10]. In previous observational studies, intestinal dysbacteriosis has been observed in both animals and patients with CNP [9, 10]; however, existing studies have not investigated the causal relationship between intestinal microbiota and prostatitis, nor have they focused on the exact role of specific intestinal microbiota taxa on prostatitis. As far as we know, this is the first Mendelian randomization study to investigate the possible causal connection between gut microbiota and prostatitis. Our findings imply a causal link between certain gut microbes and prostatitis.

Researchers have gradually looked into how the gut microbiota may play a role in the progression of prostatitis disease. Evidence was presented by Corey M. Porter et al. that suggests the human microbiome, which is found in the urinary tract, gastrointestinal tract, oral cavity, etc., may play a significant role in the health and disease of the prostate [28]. Similarly, chronic abacterial prostatitis in rats has been improved by pachymaran metabolites fermented by gut microbiota [29]. By reducing oxidative stress, controlling hormone production, changing gut microbiota, and altering DNA methylation, pachymaran was shown to reduce chronic abacterial prostatitis in another study [30]. Junsheng Liu et al.‘s additional experimental findings imply that PPs repair the gut microbiota by targeting the NK4A214 group of *Ruminococcaceae*, a separate mechanism from finasteride’s attenuation of CNP, which offers a therapeutic target for the therapy of CNP [8]. These findings imply that disruption of the gut microbiota has a role in developing prostatitis. These investigations offer crucial information for comprehending the gut microbiota’s probable function in the prostatitis condition’s progression. More research is required to fully comprehend the existence of gut microbiota dysregulation, characterized by decreased microbial diversity and changes in particular microbial taxa.

Our work successfully identified a portion of the gut microbiota that may promote or prevent prostatitis by MR analyses of two samples. A large number of analyses were performed to test the results, which showed a possible causal relationship between gut microbiota and prostatitis. According to our research, the risk of prostatitis may be decreased by the presence of *methanebacteria*, *methanobacteraceae*, *erysipelatoclostridium*, *parasutterella*. The human intestinal *Slackia* bacteria is capable of producing equol, according to another study [31], and clinical studies have shown that polyphenols like equol, including equol, have some value in treating the symptoms of patients with prostatic illnesses [32]. Furthermore, the metabolites of intestinal bacteria like *Parasutterella*, such as PPs 7-keto deoxycholic acid and haloperidol glucuronide, may function as signaling molecules of the “gut-prostate axis” and may be necessary for CNP remission in rats [29]. All of these findings are consistent with our conclusions.

*Faecalibacterium*, *LachnospiraceaeUCG004*, *Sutterella*, and *Gastranaerophilales* play a causal role in promoting the development of prostatitis. Our findings are supported by reports that members of the genus *Sutterella* are common symbionts with the ability to adhere to intestinal epithelial cells and have a role in preserving intestinal barrier function and mild pro-inflammatory capability in vitro [33]. Through the influence of their metabolites, some gut microbiota, such as *Gastranaerophilales*, may develop into novel modulators of autoimmune illnesses and contribute to a better understanding of the function that gut microbiota plays in microbiota/immune communication [34]. The most significant butyric acid producer in the human colon is *Faecalibacterium*, one of the primary elements of the gut microbiota. Contrary to what was found in our study, which demonstrated the impact of *Faecalibacterium* on prostatitis, this commensal bacterium has been thought of as a biological indicator of human health. It facilitates the inflammatory process once its number changes (decreases) [35]; this offers a new perspective for future research. Last but not least, we could not locate any additional research on the link between *Erysipelatoclostridium*, *LachnospiraceaeUCG004*, or *Parasutterella* and prostatitis, which provided new directions for our subsequent studies.

Our results close the information gap on whether the gut microbiome influences prostatitis and which taxa can hasten or prevent its onset. However, there are also certain restrictions. First, the generalization of our results to other ethnic groups may be constrained because GWAS participants are mainly of European descent. Second, we sought a unidirectional influence of 196 microbial taxa types on prostatitis because our study aimed to clarify risk factors for prostatitis to accomplish a thorough clinical intervention and lower morbidity. Third, this study did not adequately explore the precise mechanism by which the gut microbiome discussed above affects the risk of prostatitis. Fourth, we did not distinguish between acute and chronic prostatitis and separately investigated differences in gut microbiota. Fifth, further experiments are needed to validate our findings of causal links in the future. Despite these possible drawbacks, sensitivity studies showed that the causal estimates of this study are relatively reliable, they appropriately reflect the causal relationship between gut microbiota and the risk of prostatitis.

## Conclusion

In conclusion, we evaluated the causal relationship between the gut microbiome and prostatitis and identified probable causative microbial taxa for prostatitis utilizing MR analysis of two samples using publically available GWAS abstract data. This study may provide clues to the pathogenesis and novel treatments of CNP.

### Electronic supplementary material

Below is the link to the electronic supplementary material.

Supplementary Material 1

Supplementary Material 2

Supplementary Material 3

Supplementary Material 4

## Data Availability

The datasets analysed during the current study are available in the MiBioGen [https://mibiogen.gcc.rug.nl] and the IEU Open GWAS repository(https://gwas.mrcieu.ac.uk/).
