# Supplementary material for: Preliminary study of the effect of gut microbiota on the development of prostatitis
Source: BMC Med Genomics. 2024 Jan 25;17:35. doi: 10.1186/s12920-024-01812-y (PMC10809527; doi:10.1186/s12920-024-01812-y)
Supplement: Supplementary file 1 — Supplementary Material 1 [file 12920_2024_1812_MOESM1_ESM.doc]

**
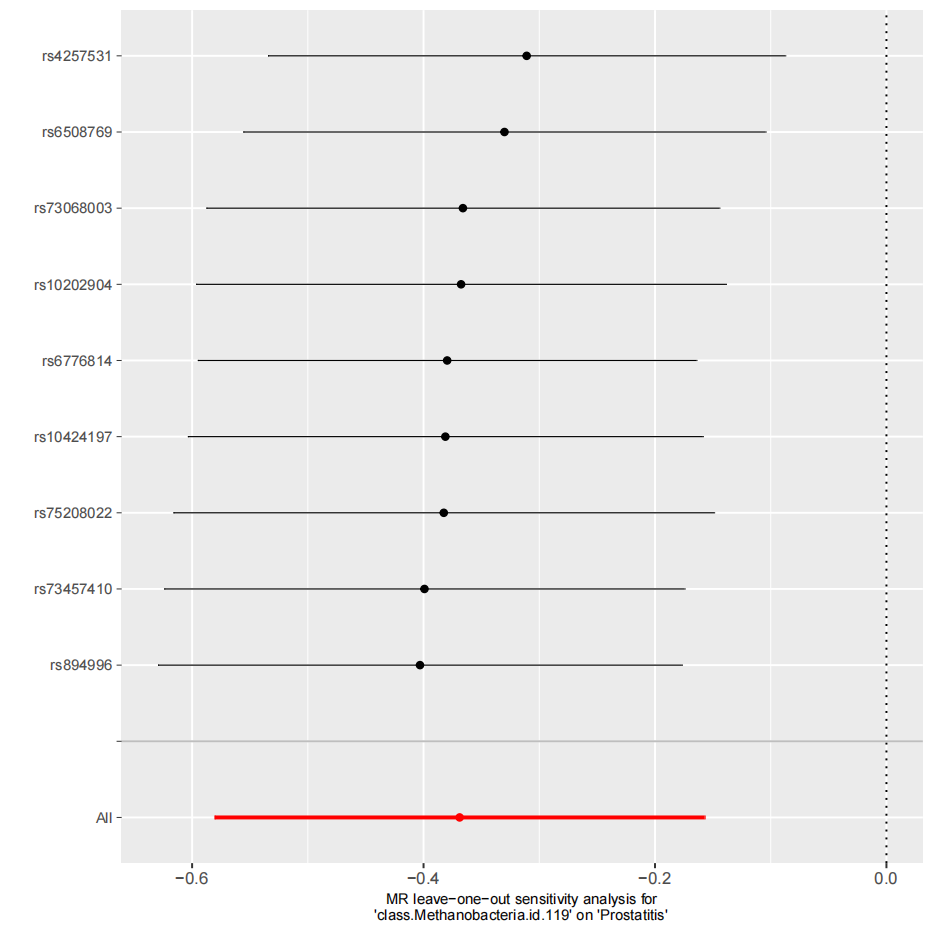

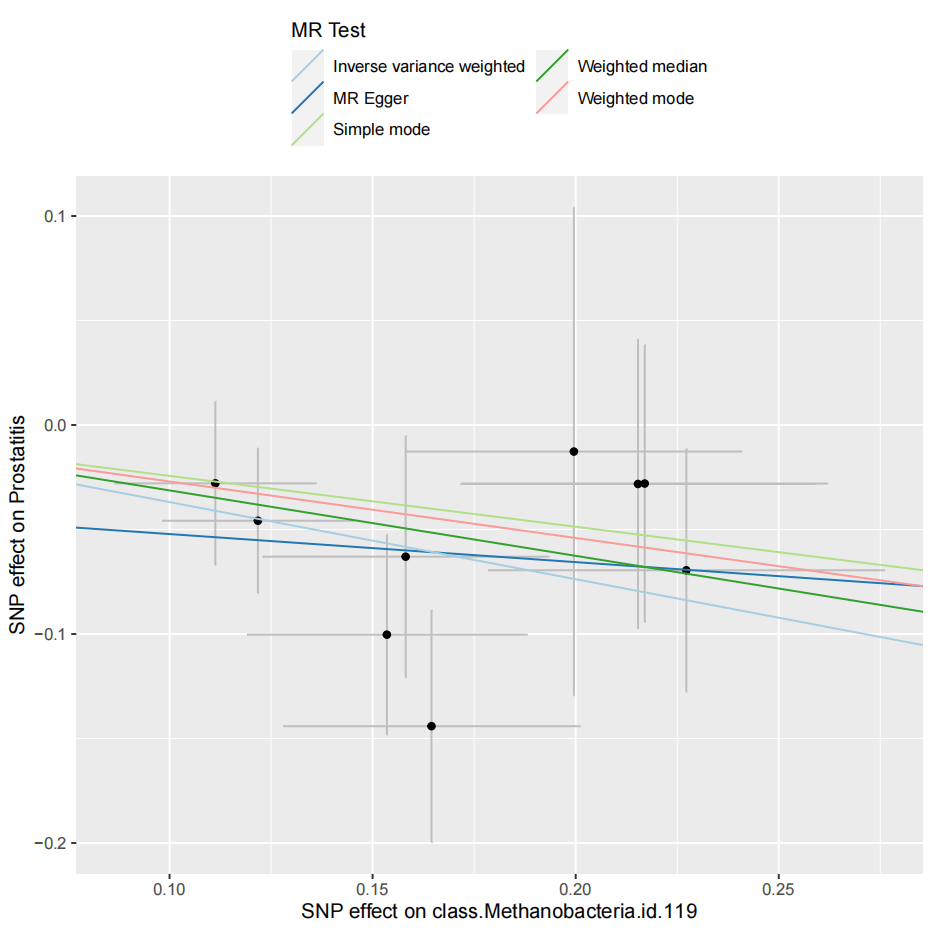
**

**Figure S1.** (A) Leave-one-out sensitivity analysis for class *Methanobacteria.id.119* on Prostatitis. (B) Scatter plots

for effect sizes of SNPs for class *Methanobacteria.id.119* on Prostatitis. Abbreviations: SNP, single nucleotide

polymorphism; MR-PRESSO, Mendelian randomization.


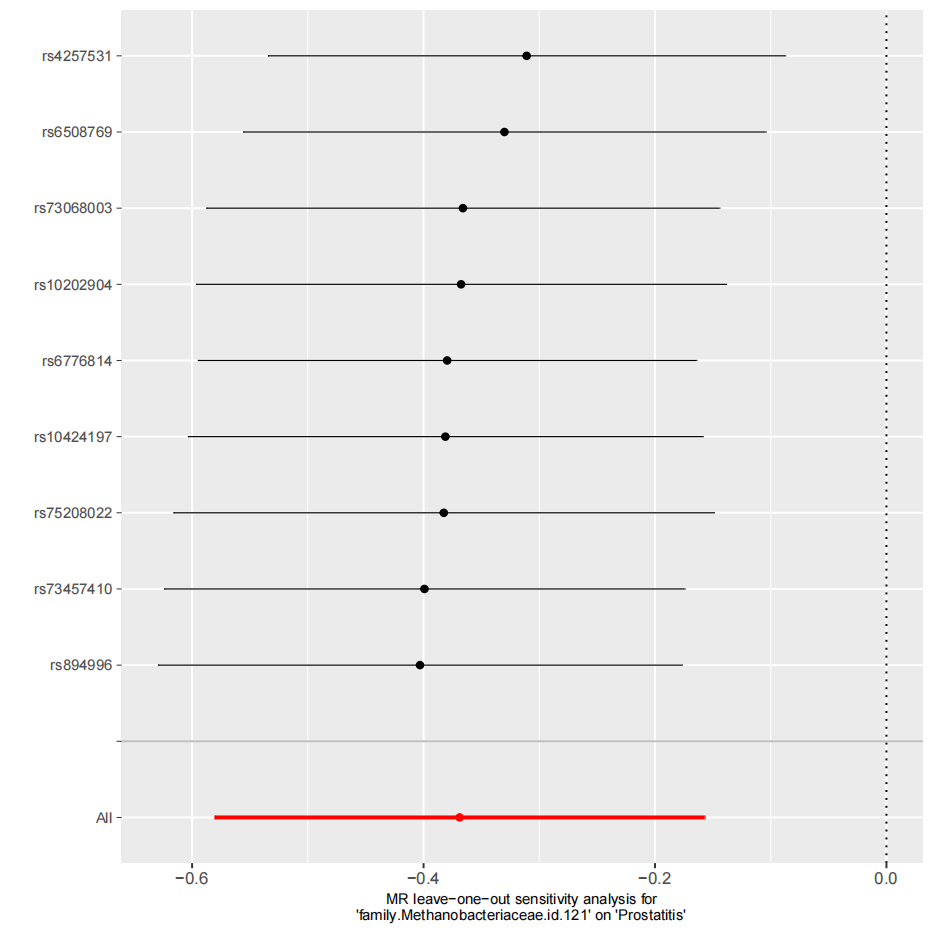

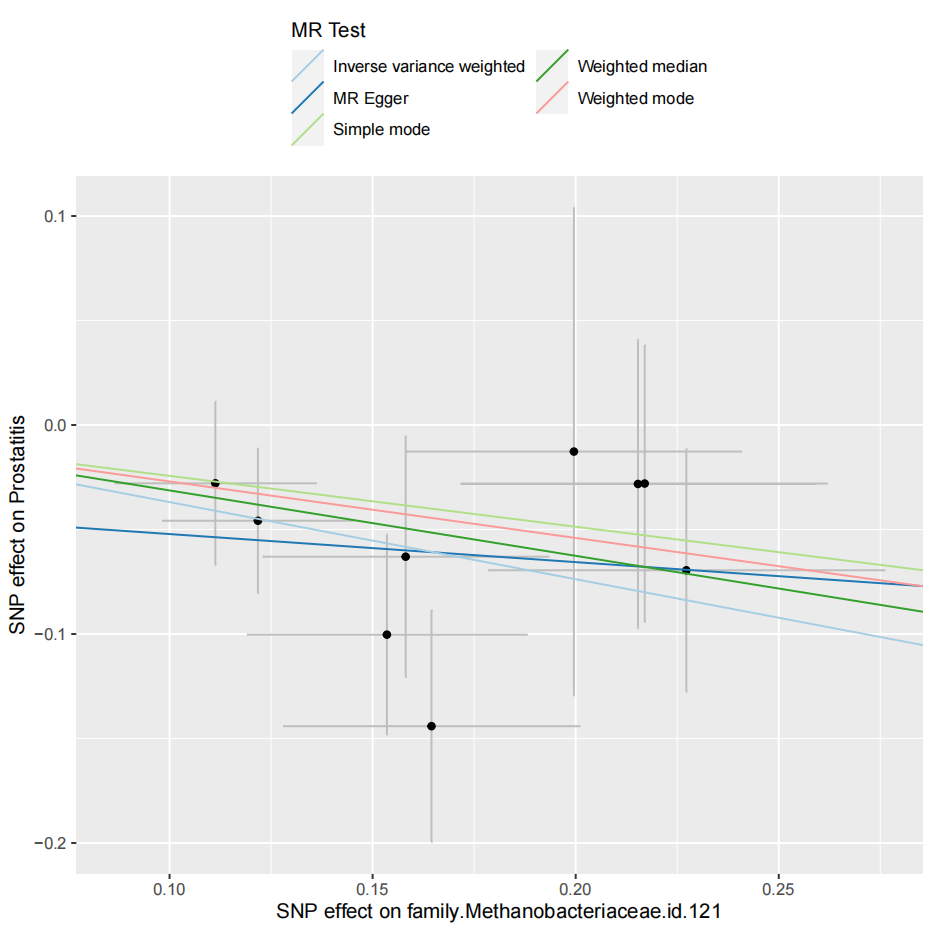


**Figure S2.** (A) Leave-one-out sensitivity analysis for *family.Methanobacteriaceae.id.121* on Prostatitis. (B) Scatter plots

for effect sizes of SNPs for *family.Methanobacteriaceae.id.121* on Prostatitis. Abbreviations: SNP, single nucleotide

polymorphism; MR-PRESSO, Mendelian randomization.


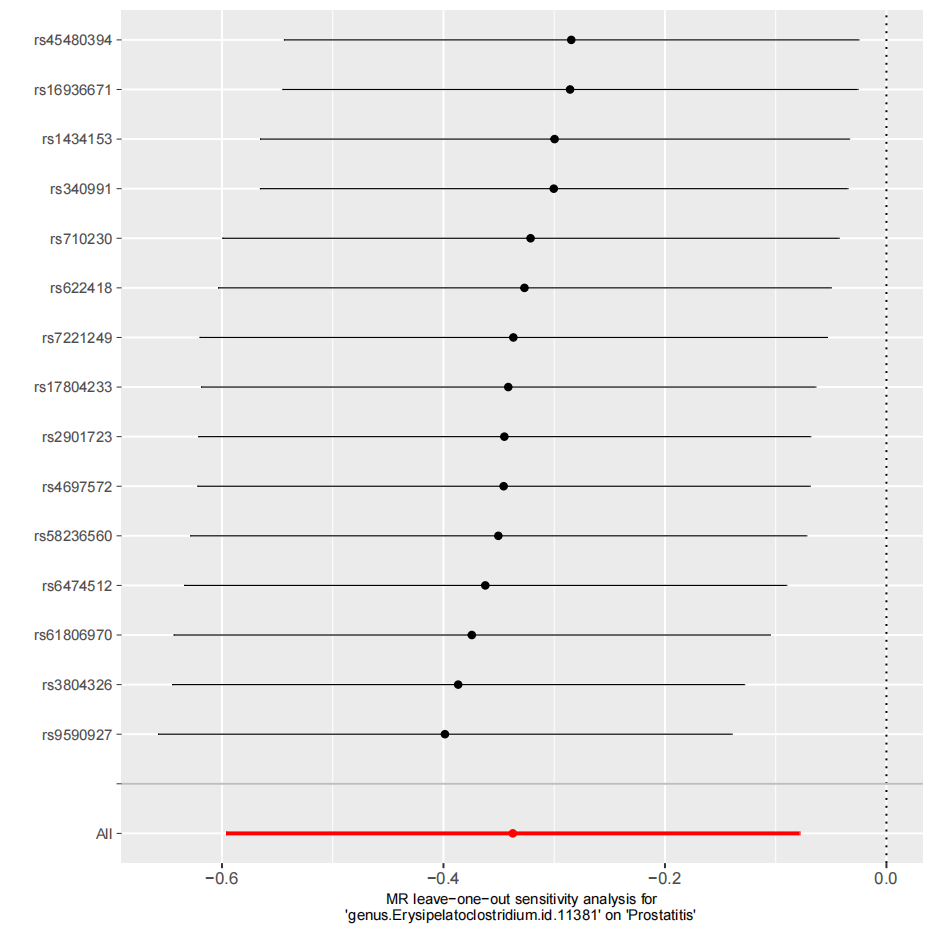

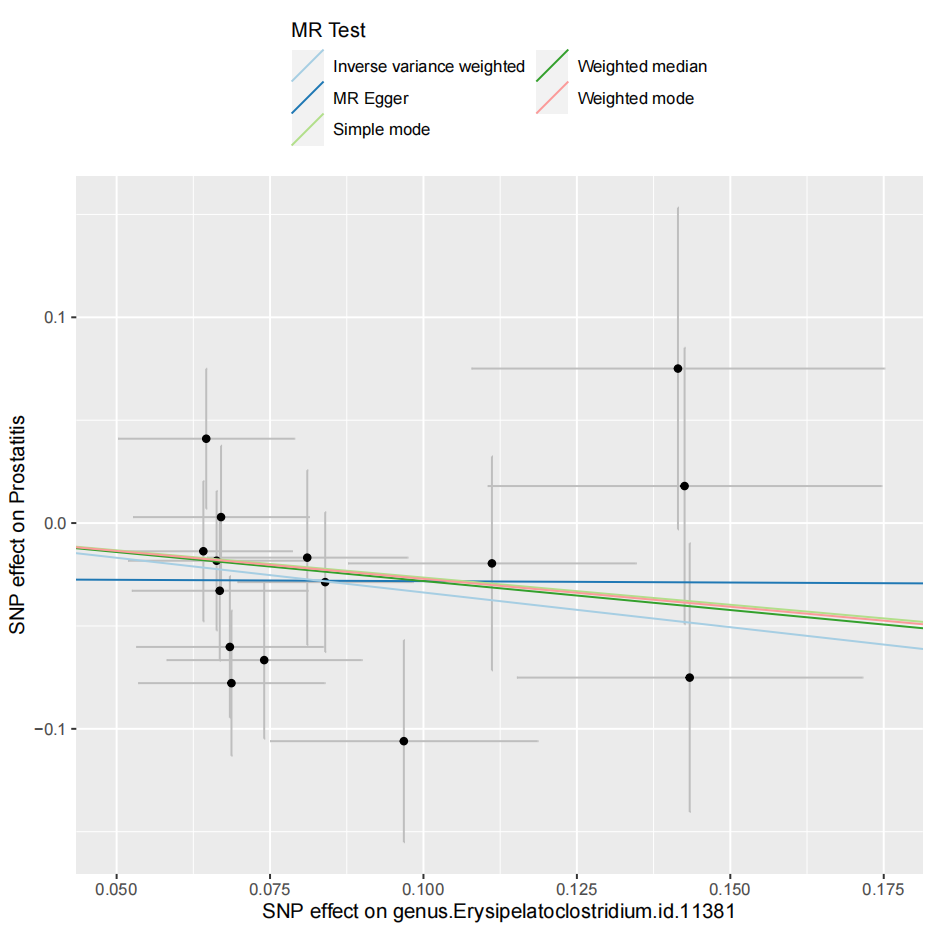


**Figure S3.** (A) Leave-one-out sensitivity analysis for *genus. Erysipelatoclostridium.id.11381* on Prostatitis. (B) Scatter plots

for effect sizes of SNPs for *genus.Erysipelatoclostridium.id.11381* on Prostatitis. Abbreviations: SNP, single nucleotide

polymorphism; MR-PRESSO, Mendelian randomization.


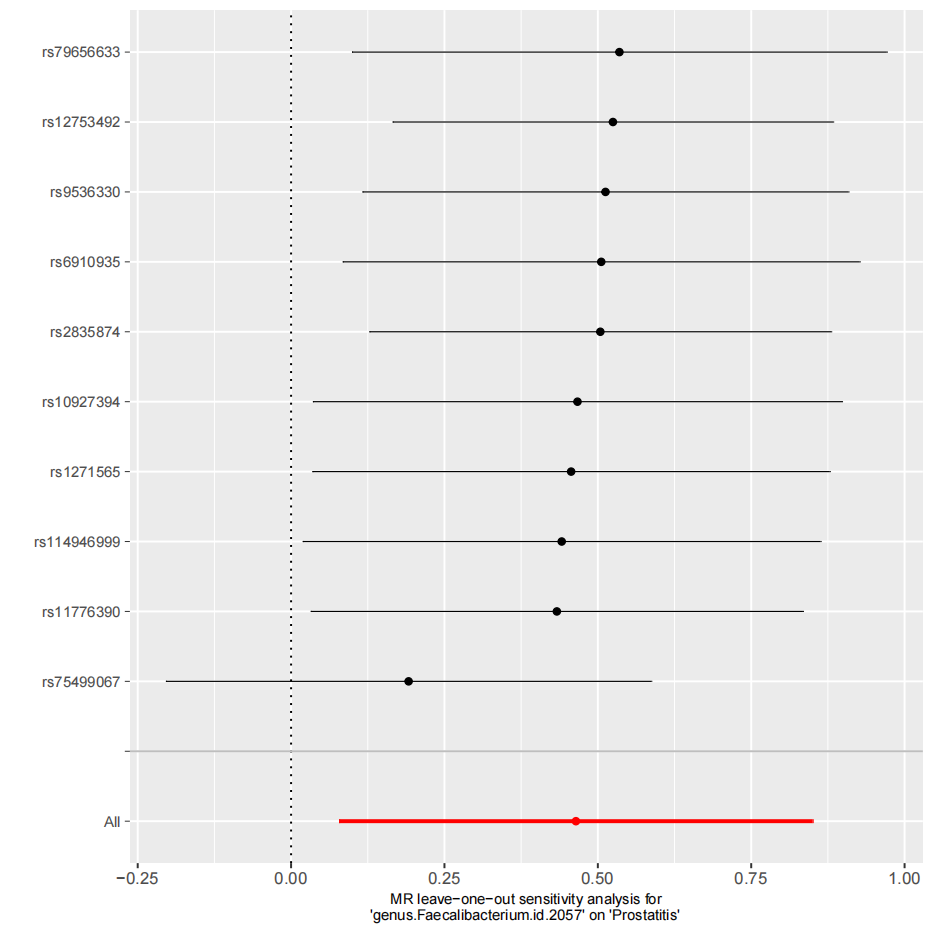

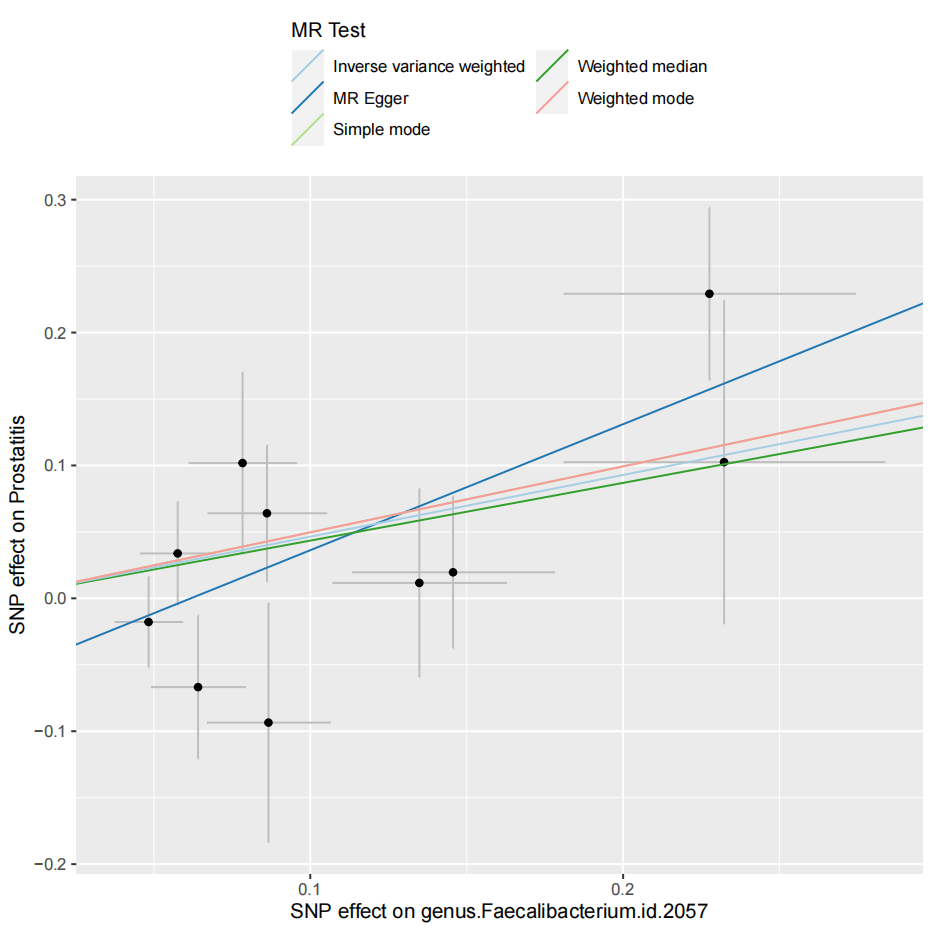


**Figure S4.** (A) Leave-one-out sensitivity analysis for *genus.Faecalibacterium.id.2057* on Prostatitis. (B) Scatter plots

for effect sizes of SNPs for *genus.Faecalibacterium.id.2057* on Prostatitis. Abbreviations: SNP, single nucleotide

polymorphism; MR-PRESSO, Mendelian randomization.


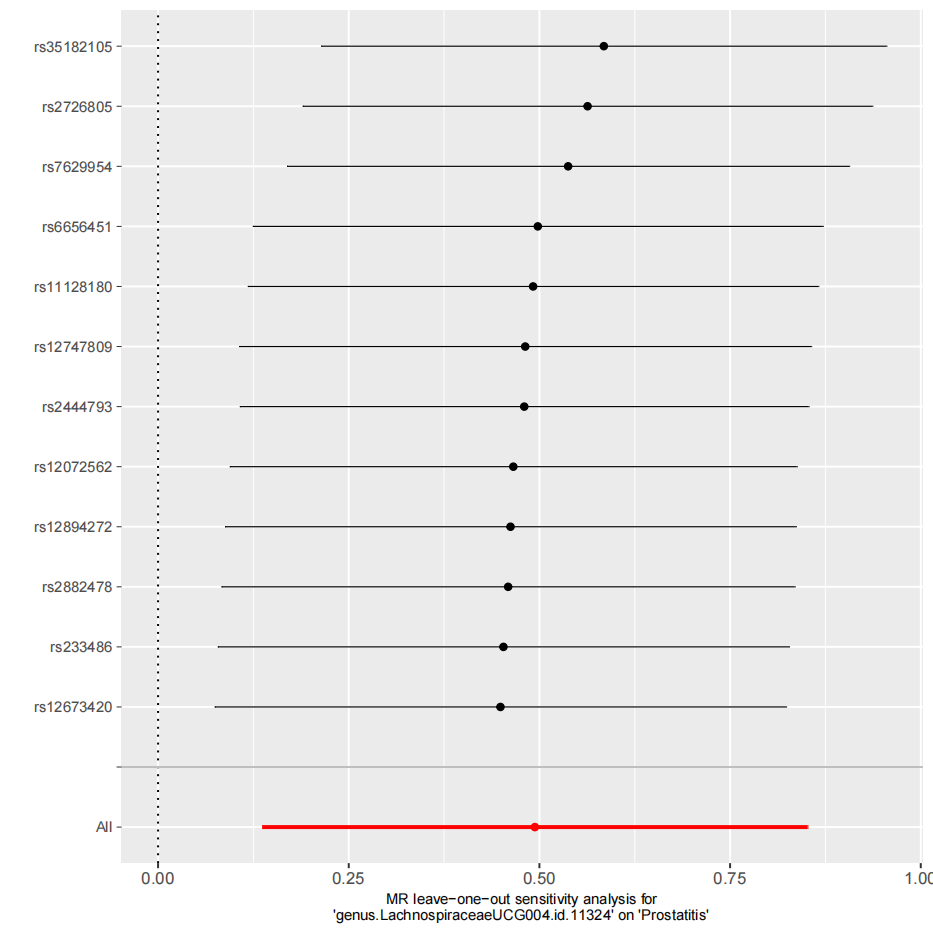

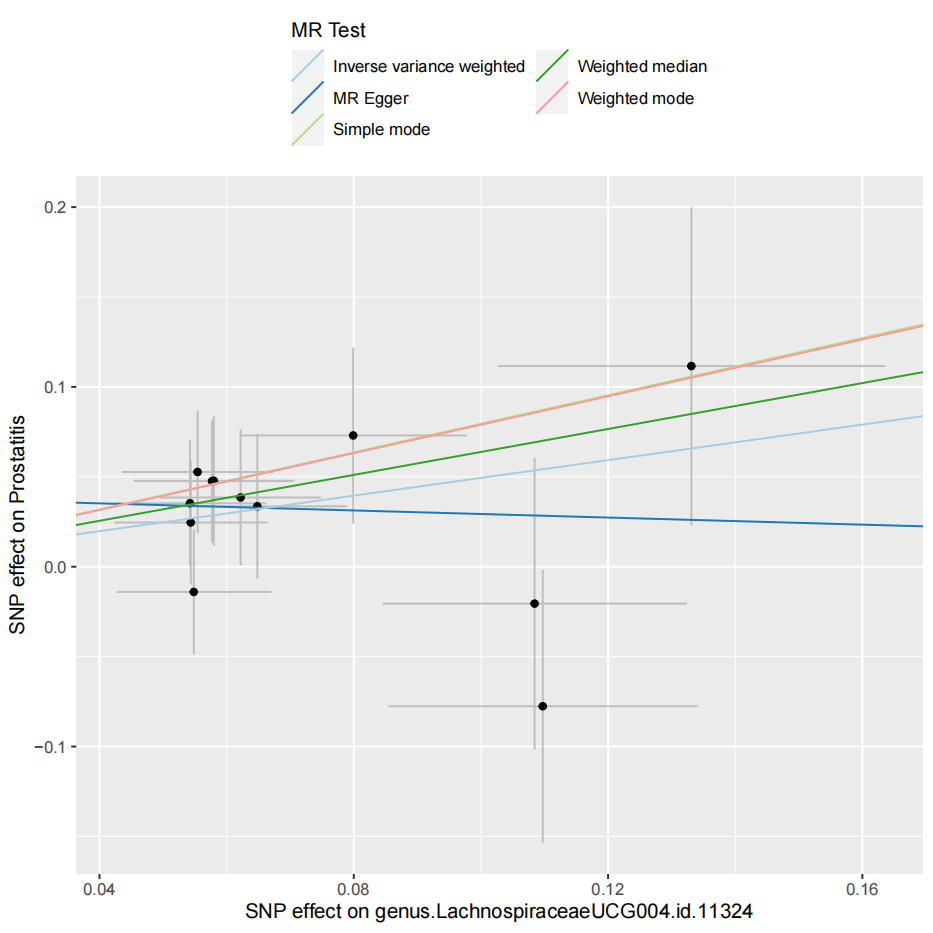


**Figure S5.** (A) Leave-one-out sensitivity analysis for *genus.LachnospiraceaeUCG004.id.11324* on Prostatitis. (B) Scatter plots

for effect sizes of SNPs for *genus.LachnospiraceaeUCG004.id.11324* on Prostatitis. Abbreviations: SNP, single nucleotide

polymorphism; MR-PRESSO, Mendelian randomization.


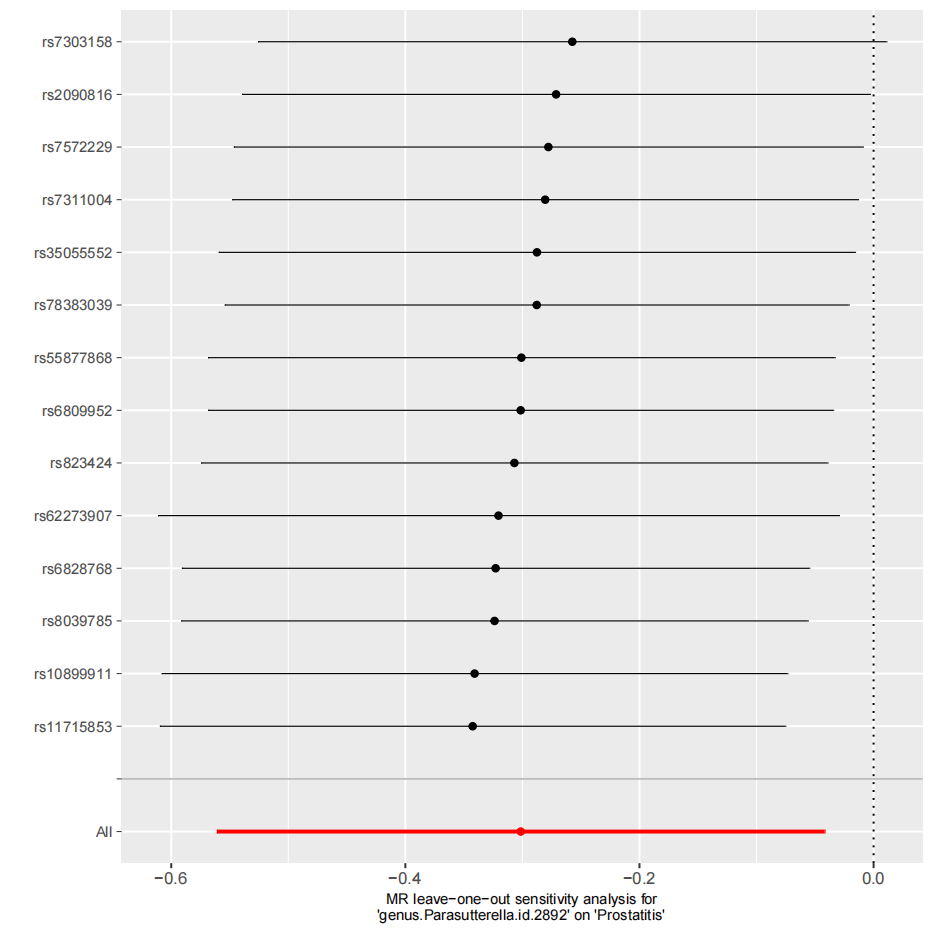

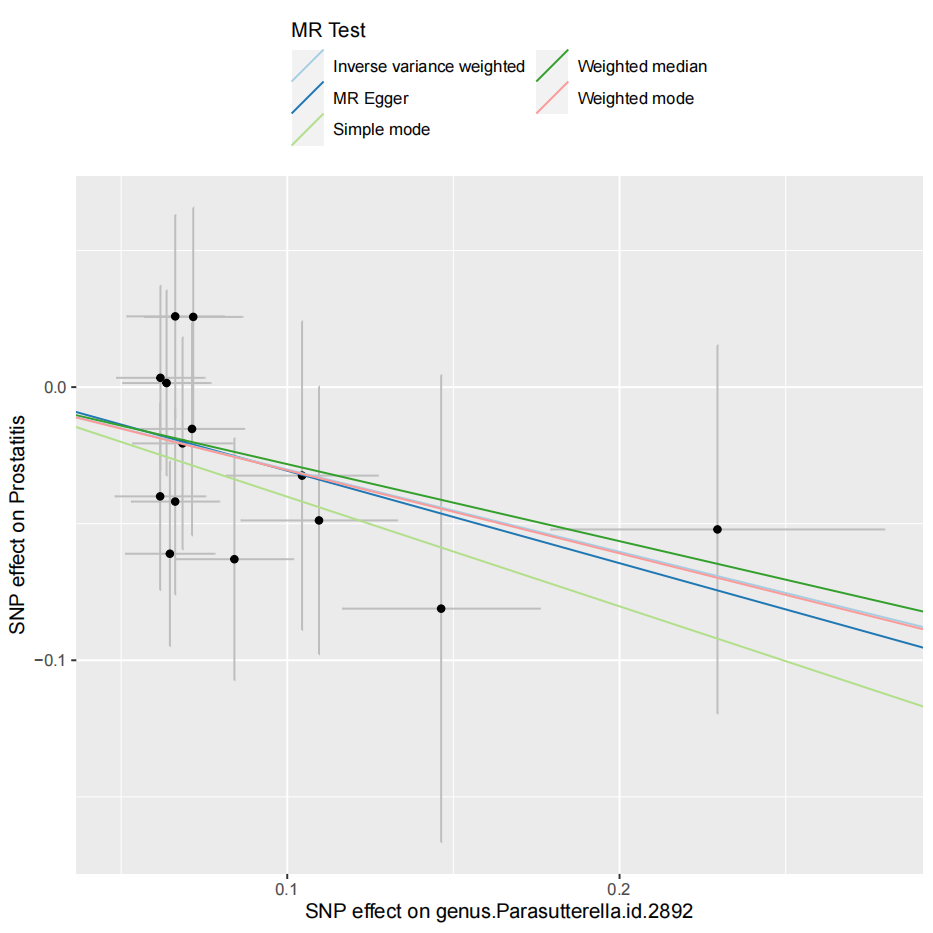


**Figure S6.** (A) Leave-one-out sensitivity analysis for *genus.Parasutterella.id.2892* on Prostatitis. (B) Scatter plots

for effect sizes of SNPs for *genus.Parasutterella.id.2892* on Prostatitis. Abbreviations: SNP, single nucleotide

polymorphism; MR-PRESSO, Mendelian randomization.

**
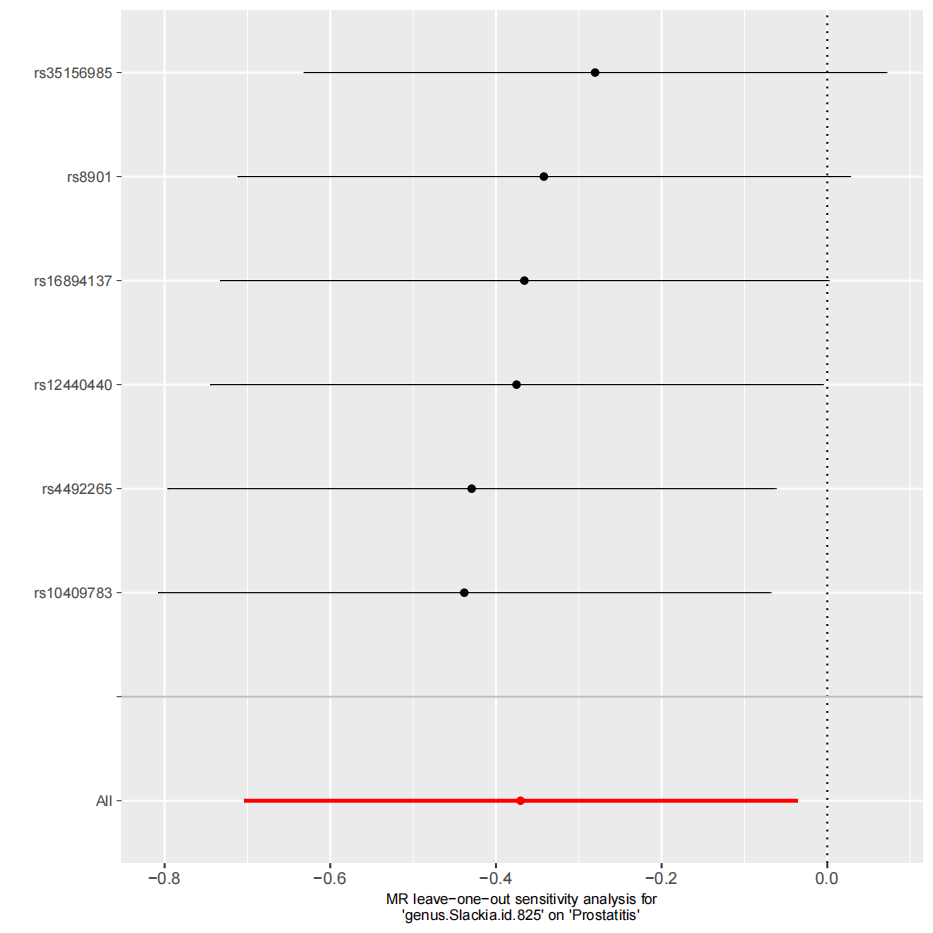

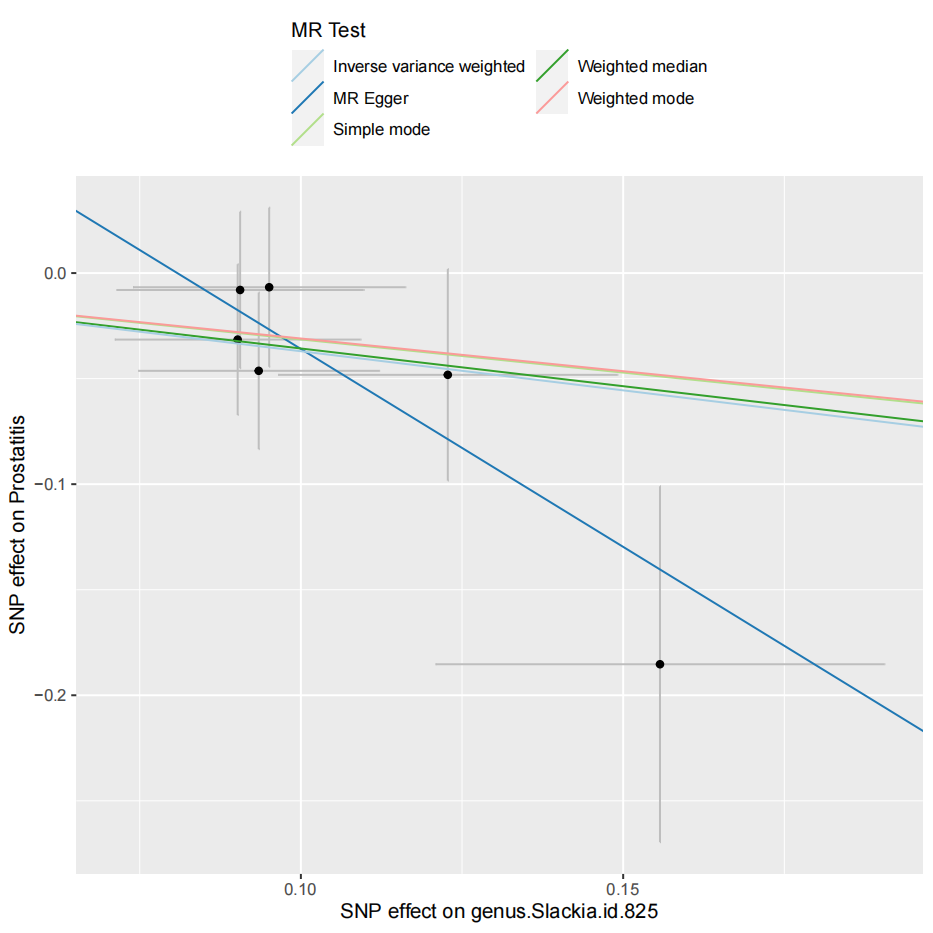
**

**Figure S7.** (A) Leave-one-out sensitivity analysis for *genus.Slackia.id.825* on Prostatitis. (B) Scatter plots

for effect sizes of SNPs for *genus.Slackia.id.825* on Prostatitis. Abbreviations: SNP, single nucleotide

polymorphism; MR-PRESSO, Mendelian randomization.


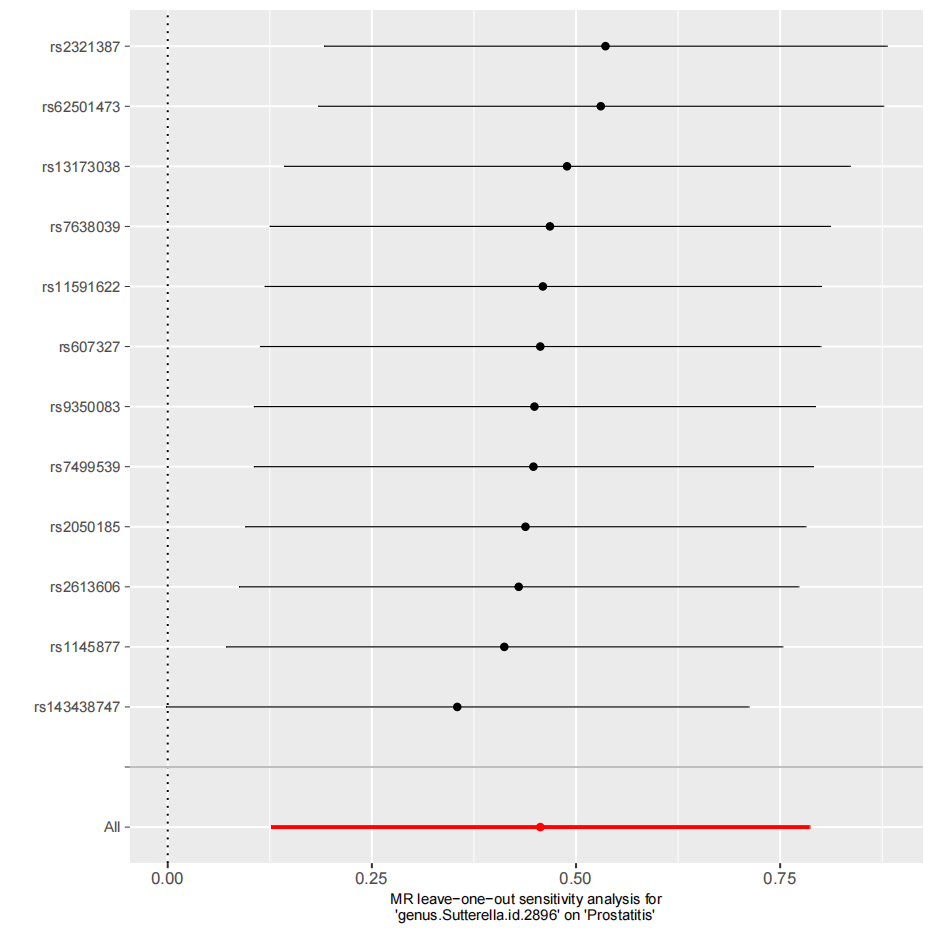

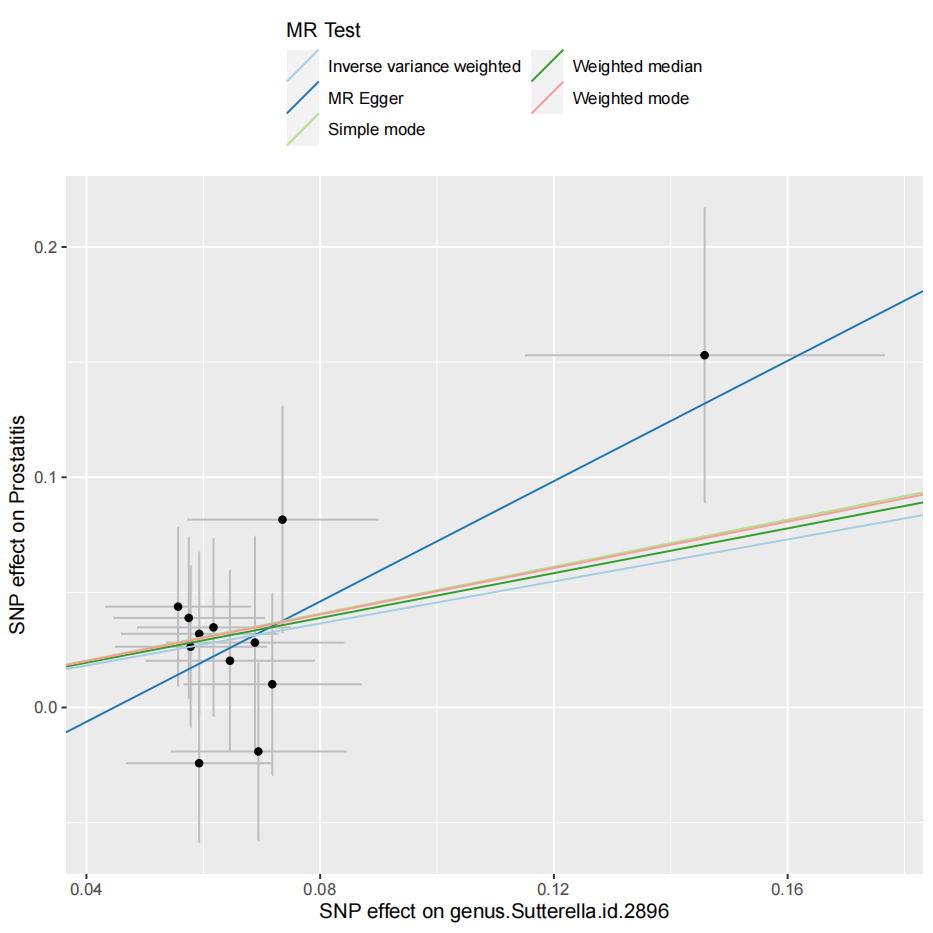


**Figure S8.** (A) Leave-one-out sensitivity analysis for *genus.Sutterella.id.2896* on Prostatitis. (B) Scatter plots

for effect sizes of SNPs for *genus.Sutterella.id.2896* on Prostatitis. Abbreviations: SNP, single nucleotide

polymorphism; MR-PRESSO, Mendelian randomization.


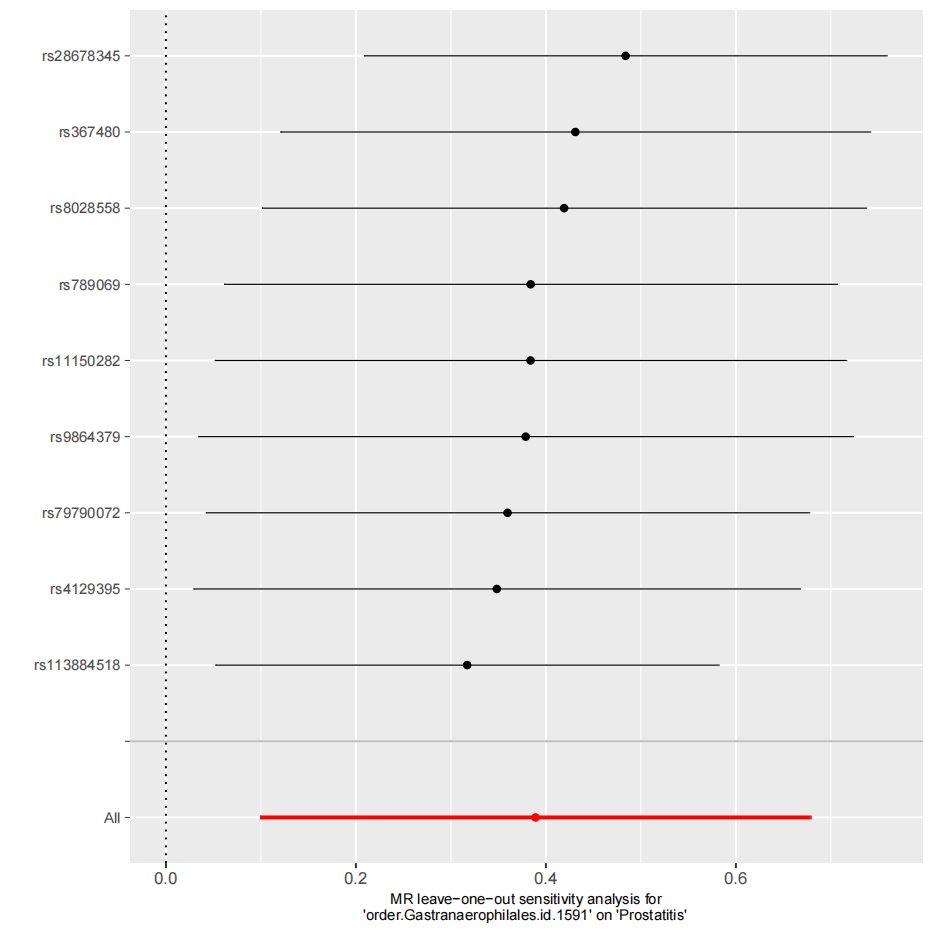

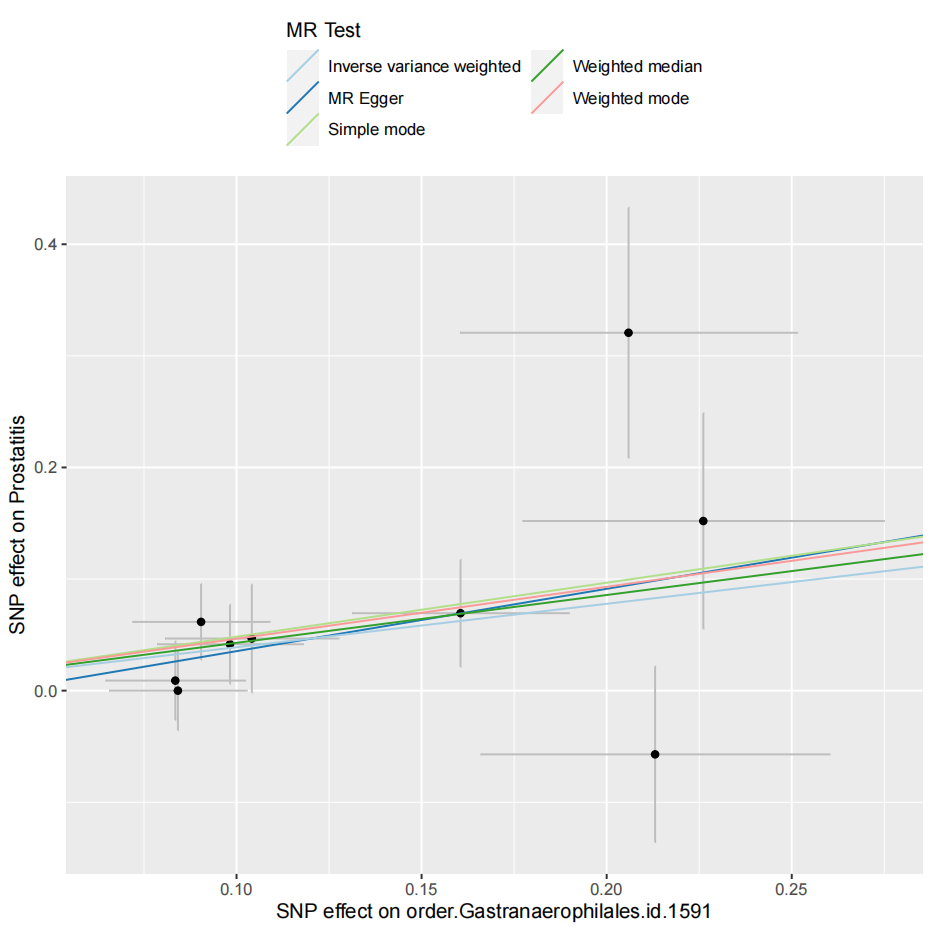


**Figure S9.** (A) Leave-one-out sensitivity analysis for *order.Gastranaerophilales.id.1591* on Prostatitis. (B) Scatter plots

for effect sizes of SNPs for *order.Gastranaerophilales.id.1591* on Prostatitis. Abbreviations: SNP, single nucleotide

polymorphism; MR-PRESSO, Mendelian randomization.
